# Script concordance test acceptability and utility for assessing medical students’ clinical reasoning: a user’s survey and an institutional prospective evaluation of students’ scores

**DOI:** 10.1186/s12909-022-03339-1

**Published:** 2022-04-13

**Authors:** Jean-Daniel Kün-Darbois, Cédric Annweiler, Nicolas Lerolle, Souhil Lebdai

**Affiliations:** 1 Maxillofacial Surgery Department, University Hospital of Angers, 49933 Angers Cedex, France; 2 Faculty for Health Sciences and Medicine, University of Angers, Angers, Angers, France; 3 Geriatric Department, University Hospital of Angers, Angers, France; 4 Intensive Care Department, University Hospital of Angers, Angers, France; 5 Urology Department, University Hospital of Angers, Angers, France

**Keywords:** Script concordance test, Evaluation, Usability and acceptability, Medical education, Clinical reasoning assessment tools, Uncertainty

## Abstract

Script Concordance Testing (SCT) is a method for clinical reasoning assessment in the field of health-care training. Our aim was to assess SCT acceptability and utility with a survey and an institutional prospective evaluation of students’ scores.With a user’s online survey, we collected the opinions and satisfaction data of all graduate students and teachers involved in the SCT setting. We performed a prospective analysis comparing the scores obtained with SCT to those obtained with the national standard evaluation modality.

General opinions about SCT were mostly negative. Students tended to express more negative opinions and perceptions. There was a lower proportion of negative responses in the teachers’ satisfaction survey. The proportion of neutral responses was higher for teachers. There was a higher proportion of positive positions towards all questions among teachers. PCC scores significantly increased each year, but SCT scores increased only between the first and second tests. PCC scores were found significantly higher than SCT scores for the second and third tests. Medical students’ and teachers’ global opinion on SCT was negative. At the beginning SCT scores were found quite similar to PCC scores. There was a higher progression for PCC scores through time.

**Supplementary Information:**

The online version contains supplementary material available at 10.1186/s12909-022-03339-1.

## Highlights

• This study is the first to evaluate students and teachers’ opinions and perceptions about SCT and to compare the SCT grades to those obtained with standard examination modalities.

• General students’ and teachers’ opinions about SCT were mostly negative.

• There was a higher progression through time for students’ scores obtained with standard examination modalities than with SCT.

**Supplementary Information:**

The online version contains supplementary material available at 10.1186/s12909-022-03339-1.

## Background

Script concordance testing (SCT) is a method used in the field of clinical reasoning assessment in health professions [1–9]. Reliability and validity of SCT in pre-graduate, graduate and post-graduate health students have been widely evaluated to date [10–12]. However, some threats to validity in the use of SCT have also been described [13]. Still, many issues surrounding SCT and their use to certify competence development have been evoked and many improvements have been proposed to date [13–16].

Uncertainty is linked to medical reasoning and one objective of medical education is to make students skilled in dealing with uncertainty [17]. SCT aims at assessing clinical reasoning under conditions of uncertainty in complex situations [5, 17]. It is designed to evaluate whether knowledge of examinees is efficiently organized for clinical actions [2]. SCT construction has been extensively described [8, 18]. A SCT begins with a short clinical scenario (vignette) which is an authentic situation in which examinees must interpret data in order to make decisions. Each scenario is followed by a series of questions that calls for judgment and reasoning about diagnostic possibilities or management options according to new elements provided by each question. It is mandatory that uncertainty, ambiguity or incompleteness are embedded in each case in order to simulate ambiguous conditions observed in real life. SCT scoring system is designed to measure the degree of concordance between examinees’ answers and the ones of a panel of experts. In consequence, SCT take into account the observed variability of experts’ responses to particular clinical situations. For each question, the answer provided by the greatest number of panel members (modal response) is considered as the gold standard reasoning under such circumstances. Other panel members’ answers reflect a difference of interpretation that can still be clinically valuable and worthy of partial credit depending on the number of experts who have given this answer [5, 10].

SCT are quite different from current examination modalities in French medical faculties, which consist mainly in multiple choice questions (MCQ) and progressive clinical cases (PCC).

MCQ and PCC aim at evaluating knowledge whereas SCT aim at assessing reasoning competency under uncertainty. French medical students and medical teachers are not familiar with the use of SCT which have been recently implemented in a few institutions such as the Medical School of the University of Angers, France. Thus, it seemed interesting to compare SCT standard examination modalities (PCC and MCQ) with SCT.

This was a prospective study in which all students in our Medical School were included and were followed during 3 years. The aim of this study was first: to evaluate students’ scores and their progression with an institutional prospective evaluation; then to evaluate SCT acceptability and utility for assessing medical students’ clinical reasoning using a user’s survey.

## Methods

This was a prospective study in which all students at our medical school were included with a 3-year follow-up. The aim of this study was to compare in a paired analysis the students’ scores and to evaluate their evolution through time. We also performed a survey to evaluate students’ and teachers’ adhesion to SCT, as a clinical reasoning test.

### Participants

This study was set at the Medical School of the University of Angers, France. Script concordance testing was used as a university examination modality, in combination with usual modalities of examination for third-, fourth- and fifth-year graduate medical students. All students and medical teachers involved in this SCT setting between September 2017 and January 2020 were included in the survey (3 academic years: 2017–2018, 2018–2019 and 2019–2020). The Medical teachers who were interviewed were involved as SCT designer and/or as expert panelists. We also prospectively analyzed the examination scores of all students who went through 3 successive examinations during this period: first examination or test 1 (T1) (first year of the study), second examination or test 2 (T2) (second year of the study) and third examination or test 3 (T3) (third year of the study). All SCTs that were used were structured similarly: a vignette (short clinical scenario) followed by a series of 1 or 3 questions that aimed at exploring any field in medical reasoning. All SCTs that were used had been validated beforehand by the teacher in charge of the concerned subject and the content of the examination questions and by the referent teacher responsible for the whole examination session. An example of SCT that has been used in this study is provided in the Supplementary data. For each SCT, a minimum of 15 experts were required. All students and teachers had been institutionally prepared for SCT. Teachers had a 1-h preparation conference for SCT conception and all SCT were reviewed by a referent teacher before submitting the SCT to the students. All students had a preparation conference and a training example before taking the examinations (a 2-h conference).

### Survey procedure and analysis

All the participants have been invited to access an online survey between March 1^st^ and March 15^th^, 2020. Invitations to participate to the survey were sent by e-mail (one invitation followed by 2 reminders). The survey was available through the software Microsoft Forms (License Office 365 A1 for Angers University). The design and validation of the survey was performed by all authors who were also 4 pedagogical referents in our institution. The survey is reported in Table 1. Five-item Likert scales were used for questions 1 to 20. Questions 1 to 17 assessed students’ and teachers’ opinions (Likert items: “strongly agree”, “agree”, “neutral”, “disagree” and “strongly disagree”) and questions 18 to 20 their satisfaction (Likert items: “very satisfied”, satisfied, “neutral”, “unsatisfied”, “very unsatisfied”). Questions were also divided into 4 groups: perceptions about SCT (questions 1 to 6), opinions about how should SCT be implemented and for what academic purposes (questions 7 to 14), opinion about SCT overall utility (questions 15 to 17) and satisfaction (questions 18 to 20). In order to facilitate the results overview, all answeres were also classified as “positive”, “neutral” or “negative” depending on how they were considered regarding SCT.

**Table 1:** Medical students’ and teachers’ satisfaction and opinion outcomes (Question 6 was for students only)

**Questions on perceptions about SCT**	
Q1	Did you find this modality of knowledge examination unsettling for you?
Q2	Did you find this modality of knowledge examination stimulating for you?
Q3	Did you find this modality of knowledge examination simple for you?
Q4	Did you have difficulty understanding the questions asked in the SCT?
Q5	Have you experienced more difficulties with this exam modality than with usual examination methods?
Q6	Regarding your scores on these SCT, have you felt that they represent well your level of skills / knowledge as you estimate it (self-perceived level)?
**Questions on opinion about how should SCT be implemented and for what academic purpose**	
Q7	In your opinion, are SCT a relevant tool in medical student’s education to improve learning?
Q8	In your opinion, are SCT a relevant tool for graded certificational evaluation of medical students to pass faculty exams?
Q9	In your opinion, are SCT a relevant tool for graded and ranked certificational evaluation of medical students to pass selection exams during medical studies?
Q10	In your opinion, should SCT have an important place in the training and knowledge examination of undergraduate medical students?
Q11	In your opinion, should SCT have an important place in the training and knowledge examination of graduate medical students?
Q12	In your opinion, should CST have an important place in the training and knowledge examination of postgraduate medical students (residents)?
Q13	In your opinion, should CST have an important place in the training and knowledge examination in initial medical education (undergraduate, graduate and postgraduate students)?
Q14	In your opinion, should CST have an important place in the training and knowledge examination in continuing medical education (regular training of doctors already in practice)?
**Questions on opinion about the overall utility of SCT to medical formation**	
Q15	In your opinion, do CST give an accurate view of clinical skills?
Q16	In your opinion, would an increased use of CST allow to get better-trained doctors?
Q17	In your opinion, would an increased use of CST allow graduate medical students to be better prepared to residency?
**Questions on satisfaction after local SCT setting**	
Q18	What was your overall level of satisfaction following the set-up of CST at the Angers Health Faculty?
Q19	What was your overall level of satisfaction regarding the principle (or substance) of CST following the set-up of CST at the Angers Health Faculty?
Q20	What was your overall level of satisfaction regarding the practical organizational procedures of CST following the set-up of CST at the Angers Health Faculty?
**Open question to collect teachers and students opinions**	
Q21	You may now express below any comment that would like to tell about CST

A qualitative evaluation was also performed to document the opinion of students and teachers. Question 21 was an optional open question that intended to gather comments which were not addressed by the survey. Response to question 21 was not mandatory. The original version of the survey was in French. It is available as supplementary data.

### Comparative analysis of examination results before and after SCT setting

This was a prospective study in which all students at our Medical School were included with a 3-year follow-up. The aim of this study was to compare in a paired analysis the students’ scores through time.

All students were evaluated at the end of each semester with a standard examination: 4 to 5 SCT and 4 to 5 progressive clinical cases (PCC) which included 15 multiple choices questions (MCQ) (standard examination modality in French Medicine Faculties). All PCC, MCQ and SCT which were used in the present study were designed to be in line with the national guidelines in order to be as similar as possible to what is expected for French national *(“Examen National Classant”)* recommendations [19].

SCT scores and PCC scores were compared to each and one another for each student during the three semesters (T1, T2 and T3). The progression scores were measured for all students who went through 3 successive examinations during the study period.

### Ethics

Students’ and teachers’ participation was anonymous and voluntary. All participants were informed of their participation in the study by e-mail. No written consent was required for publication. The experimental protocol was conducted in accordance with institutional guidelines and relevant regulations.

### Statistical analysis

Statistical analysis was performed with the SPSS 15.0 Software® (IBM Corp., Armonk, NY, USA) and Systat statistical software v13 (Systat Software, Inc., San José, CA, USA). All data were expressed as means ± standard deviation. Qualitative and quantitative variables were compared using Chi-square and Mann–Whitney tests. Differences between SCT and PCC were searched for each subject and compared using a Wilcoxon test. Paired analysis testing was performed for each student. The Spearman rank correlation test was used to assess the correlation. Statistical significance was defined as a *p* < 0.05.

## Results

### Participants

596 medical students and 41 medical teachers were asked to participate to the study. The overall response rate to the survey was 33% (241/722). Students’ response rate was 33% (200/596). Teachers’ response rate was 32% (41/126). There was no significant difference between the 2 response rates (*p* = 0.953).

### Survey analysis

The results of the students’ and teachers’ opinion and satisfaction surveys are summarized in Tables 2 and 3. An overall view of the mean results of both surveys is provided in Table 4. Teachers’ and students’ general positions (opinions and perceptions) regarding all questions tended to be negative: 47% and 58%, respectively. The proportion of neutral responses for satisfaction was higher for teachers than for the students (47% vs 15%, respectively; *p* = 0.05). The overall proportion of neutral responses for each survey was similar for students and teachers (17% vs 20%, respectively; *p* = 0.844). There was a lower proportion of negative responses in the teachers’ satisfaction compared to the students’: 25% *vs* 60%; respectively (*p* = 0.046). Students were globally less satisfied (60% not satisfied) whereas teachers were globally more undecided about their satisfaction (47%). There was a higher proportion of negative positions about all questions among students (58%) than among teachers (47%) (*p* = 0.04). There was a higher proportion of positive positions about all questions among teachers (33%) than among students (25%) (*p* = 0.041).

**Table 2:** Students’ opinion and satisfaction outcomes

	**Strongly agree**	**Agree**	**Neutral**	**Disagree**	**Strongly disagree**	**Negative opinions about SCT**	**Positive opinions about SCT**
Q1	75 (37%)	83 (41%)	24 (12%)	15 (8%)	3 (2%)	158 (78%)	18 (10%)
Q2	17 (8%)	61 (30%)	23 (12%)	56 (28%)	43 (22%)	99 (50%)	78 (38%)
Q3	1 (0.5%)	9 (4.5%)	51 (25.5%)	87 (44%)	52 (26%)	139 (70%)	10 (5%)
Q4	81 (40%)	81 (40%)	19 (10%)	15 (8%)	4(2%)	162 (80%)	19 (10%)
Q5	92 (46%)	62 (31%)	26 (13%)	18 (9%)	2 (1%)	154 (77%)	20 (10%)
Q6	5 (2%)	28 (14%)	47 (23%)	65 (33%)	55 (28%)	120 (60%)	33 (16%)
Q7	21 (10%)	72 (36%)	34 (17%)	35 (18%)	38 (19%)	73 (37%)	93 (46%)
Q8	5 (2%)	31 (15%)	25 (13%)	58 (29%)	81 (41%)	139 (70%)	36 (17%)
Q9	7 (3%)	25 (12%)	19 (10%)	51 (26%)	98 (49%)	149 (75%)	32 (15%)
Q10	2 (1%)	21 (10%)	18 (9%)	79 (40%)	80 (40%)	159 (80%)	23 (11%)
Q11	14 (7%)	48 (24%)	28 (14%)	59 (29%)	51 (26%)	110 (55%)	62 (31%)
Q12	24 (12%)	79 (39%)	51 (26%)	24 (12%)	22 (11%)	46 (23%)	103 (51%)
Q13	4 (2%)	45 (22%)	48 (24%)	62 (31%)	41 (21%)	103 (52%)	49 (24%)
Q14	36 (18%)	76 (38%)	40 (20%)	23 (11%)	25 (13%)	48 (24%)	112 (56%)
Q15	5 (2%)	35 (18%)	42 (21%)	70 (35%)	48 (24%)	118 (59%)	40 (20%)
Q16	7 (3%)	31(15%)	57 (29%)	57 (29%)	48 (24%)	105 (53%)	38 (18%)
Q17	6 (3%)	64 (32%)	36 (18%)	49 (24%)	45 (23%)	94 (47%)	70 (35%)
	**Very satisfied**	**Satisfied**	**Neutral**	**Unsatisfied**	**Very Unsatisfied**	**Negative opinion about SCT**	**Positive opinion about SCT**
Q18	4 (2%)	32 (16%)	29 (14%)	83 (42%)	52 (26%)	135 (68%)	36 (18%)
Q19	6 (3%)	62 (31%)	30 (15%)	62 (31%)	40 (20%)	102 (51%)	68 (34%)
Q20	6 (3%)	38 (19%)	33 (16%)	69 (35%)	54 (27%)	123 (62%)	44 (22%)

Results are expressed in number of students and percentage

**Table 3:** Teachers’ opinion and satisfaction outcomes

	**Strongly agree**	**Agree**	**Neutral**	**Disagree**	**Strongly disagree**	**Negative opinions about SCT**	**Positive opinions about SCT**
Q1	10 (24%)	17 (42%)	5 (12%)	6 (15%)	3 (7%)	27 (66%)	9 (22%)
Q2	5 (12%)	12 (29%)	13 (32%)	7 (17%)	4 (10%)	11 (27%)	17 (42%)
Q3	0 (0%)	7 (17%)	10 (24%)	18 (44%)	6 (15%)	24 (59%)	7 (17%)
Q4	3 (7%)	14 (34%)	10 (24%)	13 (32%)	1 (3%)	17 (41%)	14 (35%)
Q5	11 (27%)	22 (54%)	3 (7%)	5 (12%)	0 (0%)	33 (81%)	5 (12%)
Q6	*na*	*na*	*na*	*na*	*na*	*na*	*na*
Q7	6 (15%)	12 (29%)	6 (15%)	10 (24%)	7 (17%)	17 (41%)	18 (44%)
Q8	2 (5%)	11 (27%)	2 (5%)	16 (39%)	10 (24%)	26 (63%)	13 (32%)
Q9	3 (7%)	7 (17%)	6 (15%)	11 (27%)	14 (34%)	25 (61%)	10 (24%)
Q10	1 (2%)	8 (20%)	0 (0%)	18 (44%)	14 (34%)	32 (78%)	9 (22%)
Q11	1 (3%)	16 (39%)	3 (7%)	12 (29%)	9 (22%)	21 (51%)	17 (42%)
Q12	12 (29%)	11 (27%)	3 (7%)	9 (22%)	6 (15%)	15 (37%)	23 (56%)
Q13	3 (7%)	13 (32%)	5 (12%)	14 (34%)	6 (15%)	20 (49%)	16 (39%)
Q14	9 (22%)	17 (41%)	3 (7%)	8 (20%)	4 (10%)	12 (30%)	26 (63%)
Q15	1 (3%)	14 (34%)	7 (17%)	14 (34%)	5 (12%)	19 (46%)	15 (37%)
Q16	0 (0%)	8 (20%)	16 (39%)	10 (24%)	7 (17%)	17 (41%)	8 (20%)
Q17	0 (0%)	16 (39%)	7 (17%)	11 (27%)	7 (17%)	18 (44%)	16 (39%)
	**Very satisfied**	**Satisfied**	**Neutral**	**Unsatisfied**	**Very Unsatisfied**	**Negative opinion about SCT**	**Positive opinion about SCT**
Q18	2 (5%)	10 (24%)	17 (42%)	9 (22%)	3 (7%)	12 (29%)	12 (29%)
Q19	1 (3%)	12 (39%)	16 (39%)	11 (27%)	1 (2%)	12 (29%)	13 (42%)
Q20	1 (2%)	8 (20%)	25 (61%)	7 (17%)	0 (0%)	7 (17%)	9 (22%)

Results are expressed in number of teachers and percentage (*na*: not applicable)

**Table 4:** Overall opinion and satisfaction outcomes

		Neutral position	*p* vs teachers	Negative position	*p* vs teachers	Positive position	*p* vs teachers
Students	All questions	34.00 ± 11.97 (17%)	0.844	116.80 ± 34.34 (58%)	0.040	49.20 ± 29.59 (25%)	0.041
	Opinion	34.58 ± 12.93 (17%)	0.219	116.23 ± 36.92 (58%)	0.201	49.17 ± 31.70 (25%)	0.063
	Satisfaction	30.66 ± 2.08 (15%)	0.05	120.00 ± 16.70 (60%)	0.046	49.33 ± 16.65 (25%)	0.658
Teachers	All questions	8.26 ± 6.48 (20%)		19.21 ± 7.17 (47%)		13.52 ± 5.41 (33%)	
	Opinion	6.18 ± 4.26 (15%)		20.87 ± 6.47 (51%)		13.93 ± 5.77 (34%)	
	Satisfaction	19.33 ± 4.93 (47%)		10.33 ± 2.88 (25%)		11.33 ± 2.08 (28%)	

Results are expressed in average of number of students and teachers and percentages Mean ± SD (%). Grey boxes indicate significant differences students *vs* teachers for percentages

### Qualitative outcomes: expressed opinions

Negative and positive comments raised by students and teachers who answered the optional open question (Q21) are summarized in Table 5. Finally, 44% of the students (88/200) and 27% of the teachers (11/41) who have effectively participated to the study have provided qualitative comments by answering question 21. Students’ and teachers’ feedbacks were globally negative as well. Fourteen negative points and five positive points were raised by students. Eight negative points and one positive point were raised by teachers. Negative points were also raised more frequently than positive ones by both students and teachers. Some points were often mentioned by both students and teachers: “SCT are confusing”, “SCT are too ambiguous” and “a too high variability exists between experts’ responses”. One teacher raised the point that “SCT prevent students from good medical reasoning”. Difficulties of technical order were also raised by some teachers, such as the difficulty to get enough experts. Another negative point raised by some students was that there may be mismatches between the expected answers between SCT and their lectures.

**Table 5:** Negative and positive elements of students’ and teachers’ feedback (question 21)

	**Students’ feedbacks**	***N (%)***
Positive points	SCT are adapted to graduate medical students	9 (10%)
	SCT are adapted to post-graduate medical students	7 (8%)
	SCT are adapted to doctors for continuing medical education	4 (4.5%)
	The principle of SCT is excellent	1 (1%)
	SCT are discriminant	1 (1%)
Positive/Negative points	SCT are interesting in theory but not in practice	21 (24%)
Negative points	Too high variability between experts ‘responses	27 (30.5%)
	SCT are too ambiguous, not clear enough	22 (24%)
	SCT are not adapted to graduate medical students	13 (15%)
	Inadequacy is felt between obtained scores and skills / knowledge	9 (10%)
	insufficient students preparation	8 (7%)
	SCT are useless	6 (6.5%)
	SCT are confusing	4 (4.5%)
	SCT are too subjective	4 (4.5%)
	Frustrating because no possibility to justify one’s answer	3 (3.5%)
	The principle of SCT is bad	3 (3.5%)
	SCT are too difficult	3 (3.5%)
	Inadequacy is felt between SCT experts answers and national referential about the subject	2 (2%)
	Lack of detailed correction	1 (1%)
	SCT are not discriminant	1 (1%)
	**Teachers’ feedbacks**	***N (%)***
Positive points	SCT are adapted to post-graduate medical students	1 (9%)
Positive/Negative points	SCT need to be developed	1 (9%)
	SCT are useful only once knowledge is acquired	1 (9%)
Negative points	Difficult to write questions	3 (27%)
	SCT are not satisfying	2 (18%)
	Too high variability between experts ‘responses	1 (9%)
	SCT are too ambiguous, not clear enough	1 (9%)
	SCT are not adapted to graduate medical students	1 (9%)
	Difficult to recruit a sufficient number of experts	1 (9%)
	SCT are confusing	1 (9%)
	SCT prevent students from good medical reasoning	1 (9%)

Results are expressed in number of students and percentage of the responding students (*n* = 88) and teachers (*n* = 11)

### Comparative analysis of examination results obtained with SCT and progressive clinical cases

**Fig. 1:**
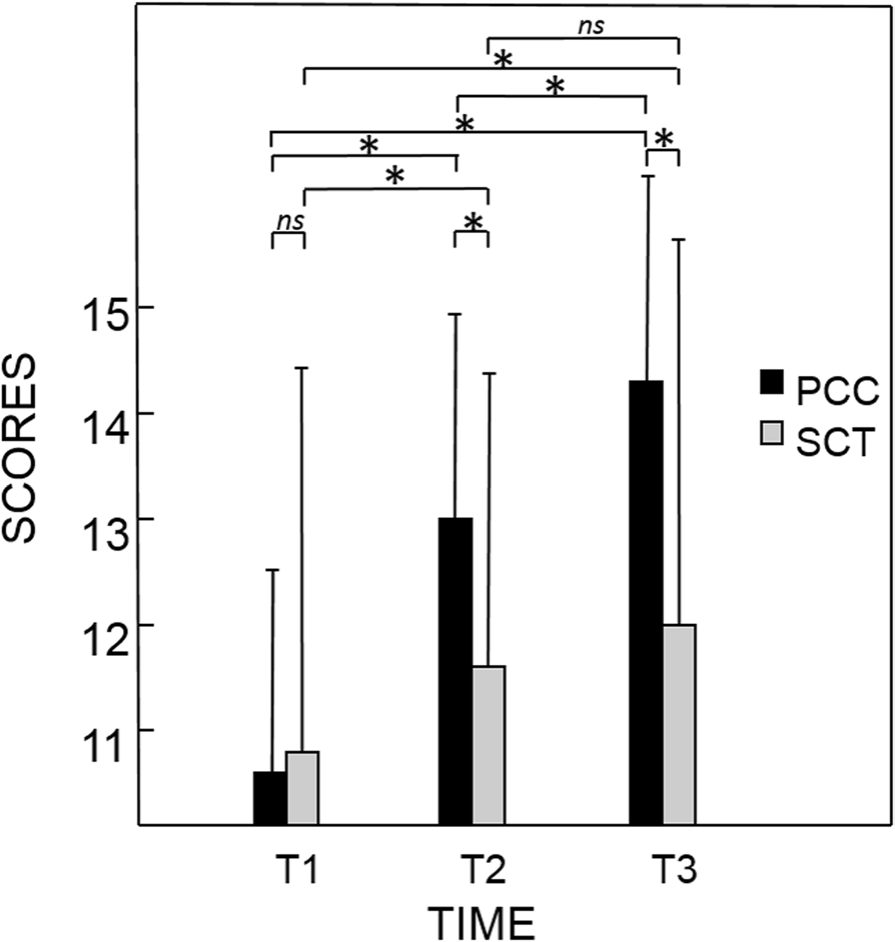
Evolution of mean scores obtained by students at Progressive Clinical Cases (■) and at Script Concordance Tests (□) expressed in absolute value out of 20 at year one (T1) year two (T2) and year three (T3) of the study

Results of comparative analysis of SCT and PCC examinations scores of students are shown in Table 6 and Fig. 1. PCC scores progressively increased each year, with a significant difference between each year (*p* < 0.001) and with a yearly mean progression of 9.25 ± 3.85 points (out of 100). On the other hand, SCT scores significantly increased only between the first and the second test (*p* = 0.004) (+ 4 points out of 100) but the difference was not significant between the second and the third test (*p* = 0.770) (+ 2 points out of 100). PCC scores were found higher than SCT scores for the second and third tests with significant differences (*p* < 0.001) (+ 7 points, + 11.5 points; respectively).

**Table 6:** Results of comparative analysis of SCT and PCC examinations scores obtained by students

		Median	Minimum	Maximum	*p vs* T1	*p vs* T2	*p* PCC *vs* SCT
T1	SCT	54	9.5	90.5	*na*	0.004	0.957
	PCC	53	5.0	74.5	*na*	< 0.001	
T2	SCT	58	27.5	91	0.004	*na*	< 0.001
	PCC	65	36.5	84.5	< 0.001	*na*	
T3	SCT	60	0.0	87.5	0.001	0.770	< 0.001
	PCC	71.5	31.5	85.5	< 0.001	< 0.001	

Scores are expressed in absolute value out of 100 (i.e. in percentage) (*na*: not applicable)

## Discussion

The response rates for the online survey were satisfactory for both teachers and students (33%, respectively). This response rate can be considered as fairly high, especially when compared to other similar studies in which reported response rates varied from 7 to 20% [20–23]. This suggests that the population of the study felt concerned by the topic. No incitement had been proposed to increase the response rate. It is also interesting to note that the response rates were the same for both students and teachers.

The present work takes place in a current context of profound changes in medical studies in France [19]. The reform of the undergraduate curriculum will be effective in 2023. The undergraduate curriculum will switch from a traditional objective-based approach to a competence-based approach. Thus, the final national examination ranking will be replaced by an evaluation system, which will assign each student a level based on three criteria: theoretical knowledge, clinical skills and the student progress training chart. Theoretical knowledge assessment will be the subject of a major diversification, with the introduction of rich context multiple choices questions (MCQ), key-feature problems (KFP) and SCT. The assessment of clinical skills will be carried out through Objective Structured Clinical Examination (OSCE). Consequently, SCT will be a mandatory new examination modality for every French medical students. It could thus be interesting to compare those standard examination modalities (PCC with MCQ) with SCT.

The existing literature has demonstrated the validity, the reliability and the feasibility of SCT at an undergraduate level and at a graduate level in order to assess clinical reasoning skills in context of uncertainty for a wide range of curricula in healthcare, [10–12]. Some threats to validity in the use of SCT have however been evoked to date [13]. However, even though SCT is now mandatory as a part of the national ranking examination and for all French undergraduate curricula, and even though medical schools have been instructed to train teachers and students for this assessment method for more than 4 years, we must admit that many French medical teachers remain unfamiliar with SCT. The results of the present study also demonstrate that fact. It seems obvious that, considering this specific French context, the topic of this article and these results warrant considerations.

These results might be explained by a distrust in innovation in an environment that has only known one kind of assessment tool such as MCQ. These opinion questionnaires might reflect the lack of training in the technique and the lack of information on the concepts underlying the evaluative process. For instance, the following aspects of SCT are critical in order to obtain a sufficient adhesion from both students and teachers: the understanding of the concept of clinical reasoning in context of uncertainty, the SCT scoring method (which no longer allows for a single correct answer), and the SCT construction method (which is diametrically different from MCQ). All these aspects are challenges to overcome in order to improve students’ and teachers’ adhesion to SCTs.

It could be interesting to find means to improve teachers’ and students’ satisfaction and adhesion to SCT. An interesting way could be the use of the recently described “evolving SCTs” (E-SCTs) which are considered by participants as more representative of real-life clinical reasoning than usual SCT [6]. In E-SCTs, the patient’s clinical history is “evolving” with thoughtful integration of new information at each stage, decisions related to clinical decision-making are then supposed to become increasingly clear [6]. Improvement in students’ training, teachers’ formation and/or organizational modalities could also be useful.

Uncertainty is linked to medical reasoning and one objective of medical education is to train students to deal with uncertainty [17]. SCT appears as a standardized, validated and reproducible tool to educate students to uncertainty in clinical practice but it is not the only one [5, 9, 17, 24]. We think that, despite controversial opinions among medical students and teachers, SCT remains an interesting tool in this field.

The present study is the first to evaluate students and teachers’ opinions and perceptions about SCT and to compare the SCT grades to those obtained with standard examination modalities (PCC). Medical students’ and teachers’ general opinions on SCT setting in our center was globally negative. There was a higher proportion of positive positions among teachers compared to students. PCC scores significantly increased each year, but SCT scores increased only between the first and second tests. PCC scores were found significantly higher than SCT scores for the second and third tests.

The neutral responses rates are globally low for both teachers and students. This fact also indicates that the population of the study felt concerned and that participants had strong opinions about SCT. However, the proportion of neutral responses in the teachers’ satisfaction part of the survey was very high, indicating that teachers were more torn than students regarding their satisfaction towards SCT setting. Almost twice more students than teachers have expressed feedbacks about SCT at question 21. Feedback were mostly negative for both teachers and students as well.

Negative perceptions and opinions about SCT users and the fact that SCT scores progress unlike traditional examinations modalities should be discussed. Regarding negative perceptions, it seems that it could mainly be linked to the novelty of SCT and to a lack of preparation of students and even teachers. Regarding the scores, those results seem positive, since they eliminate the hypothesis of an absence of correlation between students’ knowledge and their results to SCT. Thus, negative perceptions and opinions about SCT users could also be linked to insufficient teachers’ and students’ information, formation, and training about SCT.

Surprisingly, only one previous study evaluating students perceptions about SCT can be found in the international English or French literature [25]. In this study which aimed to evaluate SCT with undergraduate nursing students, it was shown that students appreciated SCT as part of a specific educational setting [25]. Since this data is lacking, we have no reference to compare our results. However, SCT seem to be largely used in Canada at any stage of medical studies [2, 11, 26, 27]. It seems to have been the case for years now. As a result, we can hypothesize that SCTs are better accepted by Canadian medical students and teachers than they are by the French. Differences exist between countries in how people or organizations deal with individual error which is highly cultural dependent [28]. Generally, a higher tolerance for mistakes is observed in North-America than in European countries such as France [29]. We hypothesize that this simple cultural difference about the perception of errors may explain teachers’ and students’ experience and opinions about SCTs.

It is important to note that the students in our study were only graduate students. No postgraduate, i.e., residents, had been solicited since SCT had not been set for postgraduate examinations. Perceptions and opinions of postgraduate students could have been different than graduate students.

We analyzed the evolution of PCC and SCT scores over 3 years. The students were initially inexperienced for both examination modalities: this is confirmed by the fact that the scores obtained with PCC and SCT were similar during the first year. Then we observed during the second and third years an increase in the scores for the two examination methods, but in different proportions. Indeed, PCC scores became significantly better than SCT scores. The gap even widens with time. Those results could appear astonishing. Performance improvement in SCT has been demonstrated in a few disciplines [30, 31]. Furthermore, it has been shown that SCT performance is correlated with clinical performance evaluations, unlike MCQs [32]. But in the same study, SCT appeared to be also initially less reliable and less preferred by students [32]. Similarly to our results, some studies reported that SCT scores also appeared correlated with those obtained on classical MCQ tests for undergraduate students [12]. In addition, recent large studies carried out within French faculties have confirmed the utility of SCT in the current context with good acceptability from the students' point of view and without any pejorative arguments from the teachers' point of view [12, 33].

A few limitations concerning the present study should be raised. At first, despite very good response rates, most of the solicited students and teachers did not answer the online survey. In consequence, a recruitment bias is a possibility considering that students and teachers that have answered the survey may have stronger opinions than the population of students and teachers that have not participated to the survey. Another limitation that could be raised is the data collection tool itself that was used. Indeed, other tools, such as focus group interviews for example, would have allowed to go more in-depth to assess the opinion and perceptions of the study participants. One last limitation of the present study is obviously its monocentric nature. Indeed, the results could have been different in other French centers, and more so in centers abroad. Despite those few limitations, the present study provides valuable data since it is the first to evaluate students’ and teachers’ opinions or perceptions about SCT and to compare the SCT grades to those obtained with standard examination modalities.

Finally, we should not give the wrong impression about SCTs. As already demonstrated in the literature SCT are a major improvement in medical education. However, our study shows that students and teachers might have some concerns during their initial experience with SCT. This does not mean that SCT are negative: it means that it is even more important to train both students and teachers and explain the importance of SCT.

## Conclusions

SCT is a recent examination modality for French medical faculties. The aim of this study was to evaluate the first three years of SCT use for faculty examinations of graduate medical students in our institution by examining students’ and teachers’ opinions and satisfaction and students’ scores evolution through time. A prospective comparison between SCT and PCC examination results was also performed.

Medical students’ and teachers’ global opinion on SCT setting in our center was globally negative. This fact may certainly be explained by the novelty of SCT setting and because of the unusual medical reasoning required. Furthermore, at the beginning, SCT scores were found quite similar to PCC scores but a higher progression for PCC scores was observed. Despite these results, SCT could be critical for medical students training especially for advanced students. According to these outcomes, actions should be taken in French medical schools in order to improve students’ and teachers’ adhesion to SCT. The use of information documents and setting-up training programs for both students and teachers might be necessary in all French medical faculties.

## Supplementary Information

**Additional file 1: Supplementary data 1.** Example of SCT used in the study.

**Additional file 2: Supplementary data 2.** Example of multiple choice questions (MCQ) that can be found in progressive clinical cases (PCC).

## Data Availability

The datasets used and analyzed for this study are available from the corresponding author on reasonable request. Raw data available as supplementary files.

## Abbreviations

MCQ: Multiple Choice Questions
PCC: Progressive Clinical Cases
SCT: Script Concordance Testing
