# Supplementary material for: Script concordance test acceptability and utility for assessing medical students’ clinical reasoning: a user’s survey and an institutional prospective evaluation of students’ scores
Source: BMC Med Educ. 2022 Apr 13;22:277. doi: 10.1186/s12909-022-03339-1 (PMC9008989; doi:10.1186/s12909-022-03339-1)
Supplement: Supplementary file 1 — Additional file 1: Supplementary data 1. Example of SCT used in the study. [file 12909_2022_3339_MOESM1_ESM.docx]

| Clinical vignette: | Mr. X fell off his bike. He complains of a chin trauma. He presents a sub-mental wound. | | | | | |
| --- | --- | --- | --- | --- | --- | --- |
| **If you were thinking of:** | **And then you find on clinical examination:** | **This hypothesis becomes:** | | | | |
| Right subcondylar mandibular fracture | Premature contact on left side | -2 | -1 | 0 | +1 | +2 |
| Left subcondylar mandibular fracture | Mandible deviation toward left side | -2 | -1 | 0 | +1 | +2 |
| Symphysis mandibular fracture | Hypoaesthesia of the lip and chin | -2 | -1 | 0 | +1 | +2 |

Scale: -2 = very unlikely, -1 = unlikely, 0 = neither likely nor unlikely, +1 = more likely, +2 = very likely

**Supplementary data 1** Example of SCT used in the study
