# Supplementary material for: Script concordance test acceptability and utility for assessing medical students’ clinical reasoning: a user’s survey and an institutional prospective evaluation of students’ scores
Source: BMC Med Educ. 2022 Apr 13;22:277. doi: 10.1186/s12909-022-03339-1 (PMC9008989; doi:10.1186/s12909-022-03339-1)
Supplement: Supplementary file 2 — Additional file 2: Supplementary data 2. Example of multiple choice questions (MCQ) that can be found in progressive clinical cases (PCC). [file 12909_2022_3339_MOESM2_ESM.docx]

| Mr. X fell off his bike. He complains of a chin trauma. He presents a sub-mental wound and a premature contact on left side. Clinical examination reveals no hypoaesthesia of the lip and chin nor intraoral bleeding or wound.  Among the following sentences, which one(s) seem(s) correct?   1. He presents signs of right subcondylar mandibular fracture 2. He presents signs of left subcondylar mandibular fracture 3. He presents signs of horizontal branch mandibular fracture 4. Radiological exam with dental panoramic radiography seems indicated to you 5. Radiological exam with facial bone X-rays seems indicated to you |
| --- |

**Supplementary data 2** Example of multiple choice questions (MCQ) that can be found in progressive clinical cases (PCC)
